# En route to sound coding strategies for optical cochlear implants

**DOI:** 10.1016/j.isci.2023.107725

**Published:** 2023-08-25

**Authors:** Lakshay Khurana, Tamas Harczos, Tobias Moser, Lukasz Jablonski

**Affiliations:** 1 Institute for Auditory Neuroscience, University Medical Center Göttingen, Göttingen, Germany; 2 Auditory Neuroscience and Optogenetics Laboratory, German Primate Center, Göttingen, Germany; 3 Auditory Neuroscience and Synaptic Nanophysiology Group, Max-Planck-Institute for Multidisciplinary Sciences, Göttingen, Germany; 4 Junior Research Group “Computational Neuroscience and Neuroengineering”, Göttingen, Germany; 5 The Doctoral Program “Sensory and Motor Neuroscience”, Göttingen Graduate Center for Neurosciences, Biophysics, and Molecular Biosciences (GGNB), Göttingen, Germany; 6 InnerEarLab, University Medical Center Göttingen, Göttingen, Germany; 7 Cluster of Excellence “Multiscale Bioimaging: from Molecular Machines to Networks of Excitable Cells” (MBExC), University of Göttingen, Göttingen, Germany

**Keywords:** Neuroscience, Bioengineering

## Abstract

Hearing loss is the most common human sensory deficit. Severe-to-complete sensorineural hearing loss is often treated by electrical cochlear implants (eCIs) bypassing dysfunctional or lost hair cells by direct stimulation of the auditory nerve. The wide current spread from each intracochlear electrode array contact activates large sets of tonotopically organized neurons limiting spectral selectivity of sound coding. Despite many efforts, an increase in the number of independent eCI stimulation channels seems impossible to achieve. Light, which can be better confined in space than electric current may help optical cochlear implants (oCIs) to overcome eCI shortcomings. In this review, we present the current state of the optogenetic sound encoding. We highlight optical sound coding strategy development capitalizing on the optical stimulation that requires fine-grained, fast, and power-efficient real-time sound processing controlling dozens of microscale optical emitters as an emerging research area.

## Graphical abstract

**Figure:**
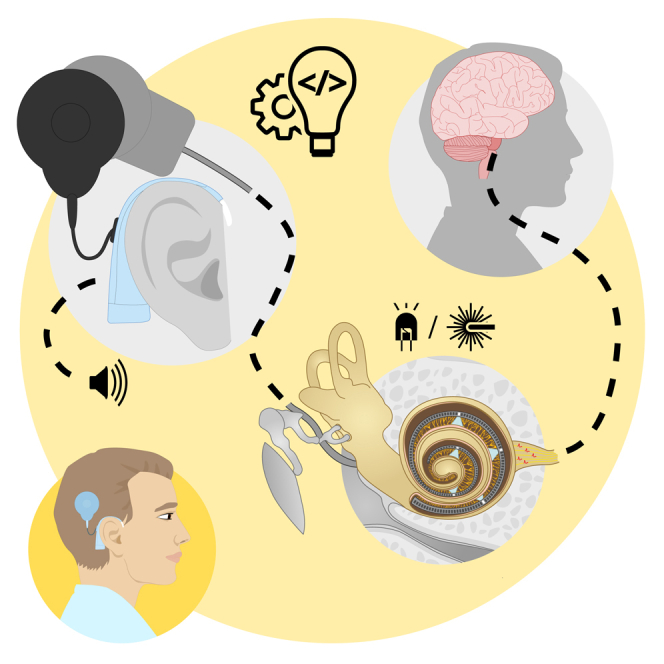

## Introduction

**Figure 1:**
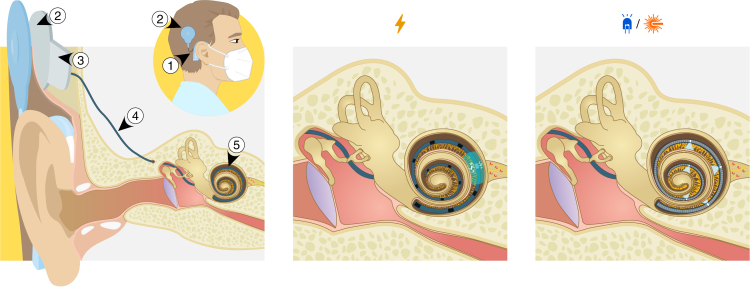
Illustration of the cochlear implant (CI) system comparing electrical CI (eCI) and optical CI (oCI) Wide spread of electric current from each of eCI electrodes is indicated comparing to confined-in-space optical stimulation with “active” or “passive” oCI. Expected increased number of perceptually independent channels of oCI and possibility to use multiple channels at very same moment of time are also depicted. Main parts of the system: (1) external behind-the-ear (BTE) sound processor, (2) inductive radio frequency (RF) link between external and internal part, (3) internal stimulator, (4) implant, (5) intracochlear array.

The World Health Organization warns of a fast growing hearing problem for years^1^ reporting, as of 2021, more than 30 million people in the world having severe to profound hearing loss and yet almost another 30 million, profound to complete.^2^ In such cases, the electrical cochlear implant (eCI) is the standard rehabilitation device associated with low implantation and failure risk.^3^^,^^4^^,^^5^ Most eCI users achieve fair open-set speech perception in the quiet. An eCI system, composed of an external sound processor and implanted stimulator, converts sound into biphasic electric current pulses stimulating, via an intracochlear electrode array, spiral ganglion neurons (SGNs) tonotopically organized along the spiral anatomy of the cochlea (Figure 1). The external processor, running the sound coding strategy, decomposes sound into frequency bands and extracts the intensity within each band. Extracted intensities serve as scaling factors for electrical pulses delivered in an interleaved fashion to electrode contacts (channels) located at the tonotopic positions corresponding to the respective frequency bands.

**Figure 2:**
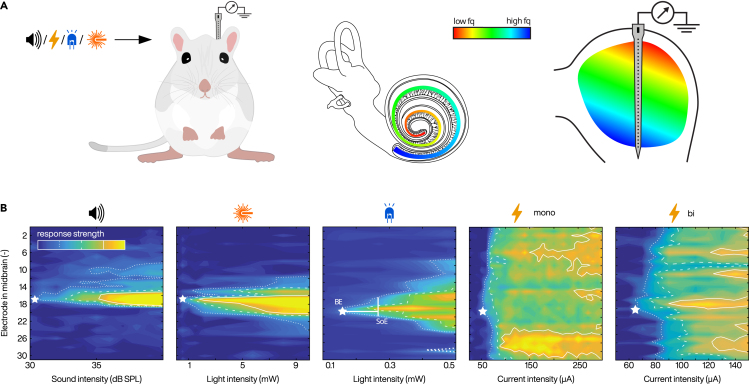
Spectral selectivity of natural acoustic hearing compared to oCI and eCI stimulation (A) Illustration of experiment where multiunit activity in response to (from left to right) acoustic, electrical, or optical stimulation of the cochlea is recorded from inferior colliculus in adult Mongolian gerbils using a multielectrode array. Tonotopic organization is color coded. (B) Assessing the cochlear spread of excitation (SoE) for (from left to right) acoustic (4 kHz, 100 ms tone burst), optical (“passive” [single waveguide, mid turn] and “active” [block of 4 LEDs, apical] oCI, 1 ms), and electrical (mono- [2nd electrode] and bipolar [2nd‒3rd electrode] eCI, 100 μs) stimulation of the gerbil cochlea by multielectrode recordings of multiunit activity (color scale and white lines) from neuronal clusters of the frequency-ordered (or tonotopically-organized) auditory midbrain. Confined spatial tuning curves indicate low SoE, i.e., high frequency selectivity of optical stimulation via waveguides or LEDs that is more similar to the acoustic stimulation than for the broad SoE of eCI. Comparison of SoE for the different modalities is based on the strength of the multiunit responses (*d’*, color code) elicited by acoustic, optical, and electrical stimulation as detected at a given midbrain electrode (asterisk indicate best electrode: i.e., electrode with lowest response threshold) and analyzed by signal detection theory measure: lines correspond to *d’* of 1.5 (small dash), 2 (large dash) and 3 (continuous). Intensity on linear axes for optical and electrical stimulation are provided for better comparability to other experiments and accessibility of energy requirements. Modified from ref.^27^^,^^28^

The electrically conductive fluid inside scala tympani of the cochlea, where the intracochlear electrode array is implanted, causes a wide spread of the electric current pulse containing information of a given frequency band for each of the 12–24 eCI electrode contacts of the array (depending on the manufacturer^6^). This leads to activation of a large fraction of SGNs (Figure 2B). As a result, spectral resolution of sound coding is often limited to less than 10 perceptually independent stimulation channels.^7^^,^^8^^,^^9^^,^^10^^,^^11^ Low spectral resolution, commonly considered a bottleneck of the eCI, limits performance in complex listening tasks (i.e., speech recognition in noisy and/or reverberant environments) and may spoil music appreciation.^12^^,^^13^^,^^14^^,^^15^ Efforts to extend the number of functional channels and reduce channel interactions are based on improving sound coding strategies and stimulators by enabling focused stimulation^16^^,^^17^^,^^18^^,^^19^^,^^20^^,^^21^^,^^22^ or current steering using multipolar stimulation (virtual channels).^23^ Alternatively, to improve neural interface, neurotrophin gene therapy increasing SGN survival and causing regeneration of spiral ganglion neurites^24^^,^^25^ or direct stimulation of the auditory nerve^26^ has been proposed. Although some of these studies have shown potential to enhance hearing experience, there remains a major clinical need for improvement.

Alternative SGN stimulation by light has the potential to overcome eCI bottlenecks (for review see ref.^29^^,^^30^^,^^31^^,^^32^). As light can be spatially confined, future optical cochlear implants (oCIs) could stimulate smaller fractions of SGNs enabling a higher number of perceptually independent stimulation channels. Two approaches to optical SGN stimulation, namely infrared direct neural stimulation (INS)^33^ and optogenetics,^34^ have been proposed. While INS concept of light stimulation via photothermal effect remains controversial for the cochlea,^35^^,^^36^^,^^37^^,^^38^ optogenetics, offering a well-defined molecular mechanism, has proved to restore auditory function in various animal deafness models in preclinical studies by several laboratories.^39^^,^^40^^,^^41^^,^^42^^,^^43^ It was shown by recordings of midbrain activity that the spectral selectivity of optogenetic SGN stimulation outperforms that of electrical stimulation^27^^,^^28^^,^^34^ (Figure 2). This conclusion was also reached for the human cochlea in computational studies investigating spread of excitation in a realistic 3D model of the cochlea^44^ in comparison to clinical electric field imaging data.^45^ Moreover, SGN recordings demonstrated that ultrafast channelrhodopsins such as Chronos, f-Chrimson, and vf-Chrimson enable optogenetic stimulation to achieve near physiological SGN firing rates^46^^,^^47^^,^^48^ (Figure 4).

In parallel, the technological implementation of the oCI progressed since the proof-of-concept study on flexible multichannel oCIs based on microscale thin-film gallium nitride (GaN) light emitting diodes (LEDs).^49^ Optimization of their light extraction and beam shaping with use of optical concentrators and micro-lenses as well as their technical characterization have been advanced^50^^,^^51^ and application in animal studies has been shown.^28^ Furthermore, studies with larger emitters^52^^,^^53^ and waveguides^54^ have been undertaken and followed by implementation, characterization, and application of a complete proof-of-concept preclinical oCI system.^55^ Such a low-weight oCI system based on a custom-made sound processor and driver can employ a dedicated real-time optical sound coding strategy taking advantage of increased number of stimulation channels and/or parallel stimulation.

**Figure 3:**
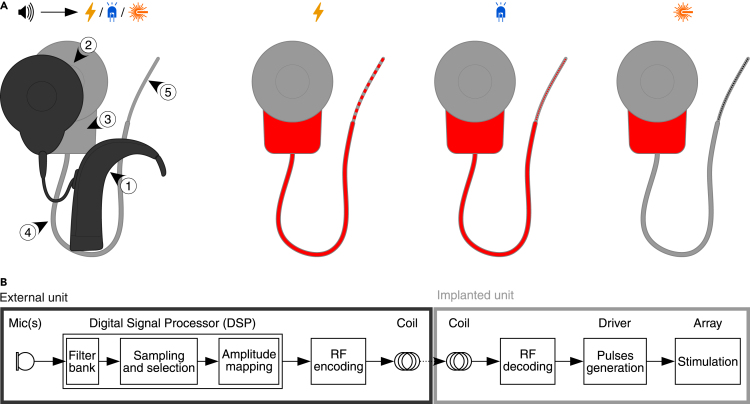
Hardware and software building blocks of CI systems (A) Illustration of contemporary two-piece design of eCI and future “active” or “passive” oCI systems showing similarities in design. Main parts of the system: (1) external behind-the-ear (BTE) sound processor, (2) magnetic radio frequency (RF) transfer link between external and internal part, (3) internal stimulator, (4) implant, (5) stimulation array. Electrically active components are highlighted in red for eCI and active (LED-based) as well as passive (waveguide-based) oCIs. (B) Scheme of sound coding into the artificial stimulation of the cochlea with CI systems.

Yet for optogenetic hearing restoration to be translated, work toward optical sound coding strategies is required in addition to preclinical development and characterization of viral gene therapy and oCI. Most currently used sound coding strategies for eCIs are based on filter-bank processing and interleaved stimulation at a constant stimulation rate (Figure 3B). In general, the conversion of sound to electric current pulses works as follows. A microphone samples the sound from surroundings at a rate fast enough for faithful reconstruction of the audible frequencies. Audio samples are processed with a filter bank to decompose the signal into frequency bands, with each band corresponding to a channel of the implant. The amplitude of each band is then extracted, e.g., by Hilbert transformation, and this determines the amplitude of the biphasic electrical pulses for the respective channel. The amplitudes are further adjusted as per patient-specific threshold and comfort levels. These pulses stimulate the SGNs in the cochlea following the place-coding principle, i.e., electrodes transmitting information on high sound frequencies stimulate SGNs toward the base of the cochlea, while those for low frequencies are placed toward the apex.

The continuous interleaved sampling (CIS)^56^ strategy was developed to tackle the problem of channel interaction, resulting from the current spread, by interleaved (non-simultaneous) stimulation. For a better transfer of temporal information, the CIS strategy uses high stimulation rates (usually about a thousand pulses per second per channel) along with short (∼100 μs) pulses and inter-pulse intervals. To increase the temporal resolution of electrical hearing even more, a number of *n*-of-*m* type strategies were developed (e.g., SMSP,^57^ SPEAK,^58^ ACE,^59^ PACE^60^ later trademarked as MP3000, TPACE,^61^ TIPS^62^). All of them are based on the principle of selecting the most significant spectral features and neglecting less important components. Sound input is filtered into *m* frequency bands and envelope information is extracted from each of them. Out of the *m* bands, *n* bands containing the largest sound pressure are used for stimulation in an interleaved manner within a given time period and at a fixed frame rate. With low *n*, the spectral representation of audio input is reduced, but the stimulation rate can be increased resulting in better temporal resolution. Conversely, for high *n*, gaining better spectral representation channel stimulation rate decreases. In general, a number of studies reported that the outcome of *n*-of-*m*-type strategies surpass those with CIS where, in each cycle, all bands are stimulated (i.e., *n* = *m*).^63^^,^^64^^,^^65^ However, regardless of the coding strategy, eCIs would need more independent channels in order to better encode spectral information. Increasing the array density would require a substantial size reduction for the single electrode contact that is limited by the maximum current density tolerated by the material. Instead, some signal processing strategies use focused stimulation and virtual channels.^16^^,^^17^^,^^18^^,^^19^^,^^20^^,^^21^^,^^22^^,^^23^ This is achieved by current steering technique where two or more channels are stimulated at different intensities to create intermediate channels between them (e.g., SpecRes strategy and its commercial version, HiRes Fidelity 120^66^). Current steering aims at improving the transfer of the original spectrum by enabling different frequencies between fixed channels. Such a strategy requires an independent current source for each electrode. Benefits for native speaking Korean users were reported.^67^ Yet in another study, only 3 out of 10 subjects improved in perception of one or more spectral cues while speech understanding in noise was not improved in any subject.^68^ A study on 65 European subjects (seven languages) demonstrated no improvement in speech understanding in any of them with HiRes Fidelity 120. Nevertheless, utilizing the increased number of perceptually independent channel predicted for the oCI might capitalize on the input stage of such a strategy and/or sound processor hardware.

Efforts to improve speech recognition include model-based coding strategies, which started with auditory-model-based ACE versions (EZ-ACE and IHC-ACE).^69^ Stimulation based on auditory modeling (SAM)^70^^,^^71^ and bio-inspired coding (BIC)^72^ strategies, implement the simulation of auditory system properties and subsequent encoding. Mimicking physiological hearing in terms of spread of excitation, cochlear delays, compression, phase locking, neural refractoriness, spike rate facilitation and adaptation, SAM and BIC, go beyond traditional strategies. They calculate each pulse individually, account for preceding stimulation results and enable individual inter-pulse intervals. These approaches could serve efforts to achieve near normal auditory percepts with optical stimulation, which, however, would require substantial computational power and increase the energy budget.

## Building blocks of the oCI system

### Implementation of optical emitter arrays

Thanks to the development in the miniaturized light-emitting, -focusing, and -delivering components, oCIs can be implemented with either an array of intracochlear optoelectronic emitters (“active oCI”, Figure 3A) or a waveguide array with extracochlear optoelectronics (“passive oCI”, Figure 3A).^73^^,^^74^ Both oCI implementations have properties, advantages and challenges to be considered when designing optical coding strategies.

Both oCI concepts have already been implemented in proof-of-concept studies in animals showing improved spectral selectivity and dynamic range over eCIs.^27^^,^^28^^,^^34^^,^^39^^,^^47^^,^^48^^,^^75^ Besides safety and stability considerations, an important emitter property is the spatial radiation pattern or intensity profile that governs the spread of neural activation in the cochlea. A Lambertian profile is characteristic of most LEDs, though the profile can be modified by use of special micro-optics, such as conical concentrators and micro-lenses.^51^ Laser-coupled waveguides, dependent on the outcoupling structure, can have a Gaussian emission profile, and therefore, spatially more confined optical stimulation is straight forward. At first glance, a narrow Gaussian profile seems optimal for oCI operation as it combines high irradiance and spatial selectivity. Yet, such emitters will need to be oriented very carefully and stably in the scala tympani, pointing directly toward the site of neural stimulation in the modiolus as even a slight shift in the orientation could substantially degrade neural excitation.^44^ Ultimately, all these factors will play a role in determining the efficiency of stimulation and these questions need to be addressed by well-constrained modeling to inform the right choice of the emitter properties. Finally, regardless of the implementation, the design might consider electrophysiological recording functionality for validation and quantification of neural excitation in analogy to eCI.

#### Active oCI

The active oCI was the first multichannel oCI that already proved its feasibility in hearing restoration in animal studies.^28^^,^^75^ This approach is very similar to the one known already from the eCI systems: wires from the driver electronics feed to the stimulation channels, which are incorporated inside a tight polymer encapsulation (Figure 3A). Already in 2014, Hernandez et al. showed promising results of optogenetic stimulation of the cochlea using an ancestor of an LED-based active oCI.^34^ Application of this single-channel device to a deaf mouse, similar to first single-channel electrical device prototype implanted in a deaf human (According to Eisen (2003), before implantation the patient had cochlea removed and only a remaining nerve stump of the auditory nerve was found where the active electrode was located. The ground electrode was embedded into the temporal muscle and monopolar configuration of stimulation was employed. As auditory nerve fibers were likely non-responsive several weeks after cochlea removal, patient hearing experience could be explained by electrical stimulation of the cochlear nucleus (next stage in the auditory pathway) making this attempt closer to a brainstem stimulator than the CI. Although the 1957 attempt of Djourno and EyriÃ¨s was not the first attempt to treat deafness with electric stimulation, it was the first avoiding stimulation of the intact cochlea eliminating the electrophonic hearing effect.) in 1957 by Djourno and Eyriès,^76^^,^^77^ paved the way for the future development of the entire concept. Also in 2014, Gossler et al., presented a proof of concept for wafer level processed μLED multichannel oCIs.^49^ Optimized for the mouse cochlea, linear arrays of μLEDs with a size of 50 × 50 μm were flip-chip-bonded on a flexible substrate carrying lines and contacts, characterized optoelectrically and inserted successfully into postmortem mouse cochleae.^49^ Since then, further developments of μLED based oCIs have been pursued^50^^,^^51^^,^^52^ and first animal studies on multichannel optogenetic stimulation using microfabricated oCIs based on miniature commercial LEDs and even smaller custom μLEDs were already presented.^28^^,^^75^ Ten-channel oCIs with the slightly bigger commercial LEDs (220 × 270 μm, C460TR2227-S2100, Cree)^52^ and addressing of individual LEDs by separate p-lines and a common n-line for all LED were characterized *in vitro* and *in vivo.*^75^ Combined with a custom-made preclinical sound processor and oCI driver circuitry, these oCIs enabled successful behavioral testing of optogenetic stimulation in rats providing the first proof of concept for a complete multichannel oCI system.^55^^,^^75^ Increasing the number of channels was achieved with smaller custom μLEDs (60 × 60 μm) and matrix addressing. There, blocks of emitters are connected with a common contact and each emitter in a block has another common contact with corresponding emitter of other blocks.^50^ Wiring is minimized but addressing is only partially independent: each μLED within a given block is independently addressable as long as only one block is selected at a given time. Employing this approach up to 144 μLEDs could be operated with 12 n-contacts and 12 p-contacts.^50^ Using μLED-based oCIs higher spectral selectivity of optogenetic stimulation compared to eCI was reported based on recordings from inferior colliculus in adult Mongolian gerbils.^28^ However, with the typical maximal radiant flux of ∼0.8 mW of the individual μLEDs only a third of them elicited significant neural responses. Therefore, the spatial selectivity was assessed by activating blocks of four neighboring μLEDs, likely underestimating the selectivity achievable with μLED implants. Increased μLED emission and/or using more potent channelrhodopsins, and/or increasing their expression in SGNs will be required for studying the spread of excitation upon stimulation by individual μLEDs. Indeed, efforts toward improved light extraction using microscale optical concentrators and lenses (10 μm in diameter) enhanced the μLED performance *in vitro.*^51^ Recently, advanced concepts of LED addressing for independent operation of a large number of channels were presented.^78^ Such a tri-state switching scheme would allow for increased number of LEDs with minimal wiring in the future implant designs.

The high-efficiency low-voltage organic LED (OLED) is another emitter candidate for the active oCI implementation. Integration of OLEDs on top of the complementary metal oxide–semiconductor substrates (OLED-on-CMOS technology)^79^ is an interesting concept for a fully integrated optoelectronic system in oCIs provided that achievable irradiance matches the requirements of neural excitation. OLED technology already offers a variety of colors (red, orange, white, green, and blue) and the CMOS architecture would enable the exact and fast control of a large number of emitter elements in arrays with a minimum number of metallic lines (daisy chaining of chip-to-chip interconnects) for mechanical flexibility of the implant.^80^ Integrated photodetection would make it possible to obtain direct feedback on the stimulus intensity for a closed-loop feedback control of the individual OLED that could serve the oCI fitting and control of operation. OLED technology was already presented to stimulate cells placed on top of the two-dimensional array as well as modulate cortical neurons.^81^^,^^82^ Nevertheless, OLED technology is yet to be tested for application in the cochlea.

Finally, microscale lasers such as vertical-cavity surface-emitting lasers (VCSELs) have been employed for active oCI^83^ that provide narrow beam profiles and should offer sufficient radiant flux for optogenetic stimulation. Still, supplying sufficient current to the laser as well as achieving a stable hermetic yet transparent and flexible encapsulation of the array remain important challenges to tackle.

#### Passive oCI

Although the active oCI implementation is currently the most technologically ready, passive oCIs represent an attractive option with a favorable safety and stability profile. In this design, all electrically active components are hermetically enclosed in the titanium housing of the implant in analogy to the current sources in eCI systems^73^ (Figure 3A). Although there is no proof-of-concept preclinical animal study with chronic multichannel implementation yet, first important development goals have already been achieved.

Light delivered from optical fibers chronically implanted into the round window for single-channel stimulation of the cochlea drove a behavioral response in awake gerbils and proved an auditory percept due to optogenetic stimulation.^39^ In a follow-up study, the spectral selectivity of the waveguide approach was addressed by recordings from inferior colliculus in response to stimulation from three independent fibers in three different regions of gerbil cochlea.^27^ Despite the fact that the fibers were placed into cochlear windows opposing the medial wall, near physiological spectral selectivity was found (Figure 2B). Such high selectivity was not observed with blocks of 4 μLEDs inserted inside scala tympani similar to the typical CI position (Figure 2B). Computational modeling studies also confirmed that Gaussian-profile emission of waveguides to be more suitable candidates than Lambertian-profile μLEDs for use in oCIs, as they achieve higher irradiance at the same radiant flux with lower spectral spread.^44^ Development of miniaturized waveguides, their coupling to miniature laser diodes, and light-outcoupling (e.g., via integrated micromirrors) represent current work in progress.^84^^,^^85^^,^^86^^,^^87^ Recent studies on fabrication and characterization of waveguide arrays for oCIs demonstrated successful implantation into the gerbil cochlea,^88^ yet functional results remain to be obtained.

### Implementation of the optical CI driver hardware

Just like for eCI, the oCI hardware design determines the features available for coding strategy. Considerations for oCI design include single vs. multiple wavelength(s), e.g., for diversified excitation or combined excitation and inhibition, as well as number, type and operation of emitters and their addressing. The latter is relevant for independent operation of channels as required for parallel stimulation of multiple sites. Power consumption is another important consideration as current estimates of required pulse energy of oCI exceed that of eCI (see below). Hence, maximizing efficiency of emitter operation such as driving laser diodes with large current but ultrashort pulses that are then integrated by optogenetically modified SGNs is important and will also serve the oCI heat management. Efficient integration of injected currents by SGNs has been demonstrated for direct current injection^89^ and optogenetic stimulation.^90^ Moreover, power-efficient optogenetic emulation of physiological sound coding with parallel stimulation should balance the transfer of spectral and intensity information. For example, optogenetic coding might consider recruiting emitters that neighbor one of the *n* channels for broadening neural excitation at a specific tonotopic position for encoding loud sounds.

**Figure 4:**
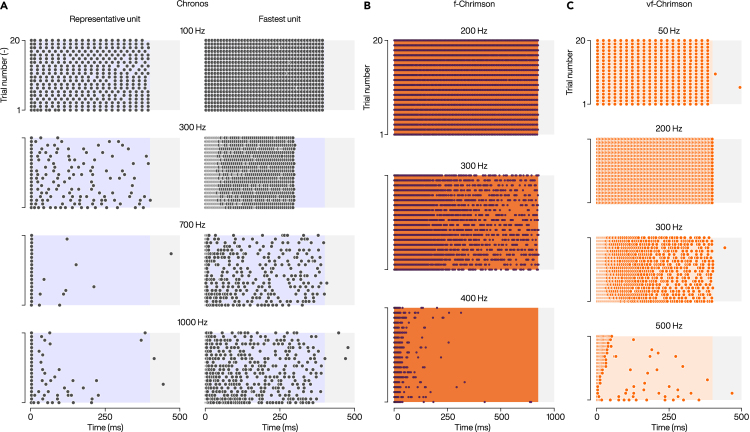
Extracellular recordings of firing rates from single putative SGNs in response to optogenetic stimulations (A) Spiking activity from a putative SGN units expressing Chronos in response to 400-ms-long trains of laser pulses (30 mW, 1ms for <700 Hz or 500 μs for ≥700 Hz) at different frequencies. (B) Activity of an SGN units expressing f-Chrimson in response to 900-ms-long trains of laser pulses (1 ms). (C) Traces from a putative SGN unit expressing vf-Chrimson in response to 400-ms-long trains of laser pulses (43 mW, 1 ms). Modified from ref.^46^^,^^47^^,^^48^

Future oCIs directly trigger firing of SGNs that express channelrhodopsins (ChR, light-gated ion channels), i.e., bypassing dysfunctional or lost IHCs. Wavelength, kinetics, and energetics of optogenetic stimulation need to be tuned to the action spectrum, light sensitivity, conductance, and gating kinetics of the ChR employed. To a first approximation the temporal fidelity of optogenetic SGN stimulation is limited by deactivation time constants of the channelrhodopsins. For example, the blue-light-activated ChR2,^91^ which was the first ChR employed for neural stimulation^92^ and is widely used in the life sciences, deactivates with the time constant of ∼10 ms at room temperature and enables firing up to ∼50 Hz in neurons.^92^ Likewise, the red-light-activated ChR Chrimson (peak response at 590 nm) has a rather long deactivation time constant of ∼25 ms at room temperature.^47^ Both ChR seemed ill-suited for the high temporal fidelity desired for optogenetic sound encoding. Therefore, efforts have been undertaken to engineer ultrafast ChRs. Chrimson variants, that, at physiological temperature, have deactivation constant of 3.2 ms (fast, f-Chrimson) and 1.6 ms (ultrafast, vf-Chrimson) have been generated^47^ and characterized.^47^^,^^48^^,^^93^ These variants enable reliable SGNs firing with good temporal precision (vector strength of at least 0.5) at stimulation rates of up to ∼200 Hz (Figure 4).^47^^,^^48^^,^^93^ Even faster deactivation constant of 0.8 ms at physiological temperature^46^ is exhibited by the ultrafast blue-light-activated Chronos (peak response at 500 nm)^94^ enabling stimulation rates beyond 200 Hz.^46^ However, the resulting shorter channel opening time provides less charge transfer per photon absorption, which in turn extends the power needed for stimulation. Therefore, oCI strategies working with currently available ChRs will likely limit the stimulation rate to 200 Hz, i.e., lower than contemporary eCI strategies. We consider it likely that potential disadvantages of temporal coding resulting from the lower stimulation rate will be offset by the enhanced spectral coding.

Ultrafast red-light-activated ChRs such as f-Chrimson and vf-Chrimson seem to be good candidates for clinical translation given suitable kinetics and the lower risk for phototoxicity for the lower energy photons. As the energy requirement of optogenetic stimulation is another important consideration and more favorable for f-Chrimson than for vf-Chrimson, f-Chrimson might be the better choice as it lends to comparable temporal fidelity of SGN firing.^47^^,^^48^ Still, the single-channel energy threshold of 0.5 μJ found by auditory brainstem response (ABR) recordings in response to 1-ms-long laser light pulse^47^ indicates that the energy requirement still exceeds that of the single-channel eCI ABR energy threshold of 0.025 μJ (calculation based on interpolation from ABR protocol with a 1-ms long train consisting of ten bipolar pulses of 45 μs phase duration resulting in a threshold of ∼60 μA^95^ and electrode impedance of 7 kΩ). Increased number of oCI channels comparing to eCI may lead to higher energy consumption. Also, influence of light source type as well as transduction efficiency on overall energy budget should be considered too (for more discussion see section on risks and issues of oCI technology). Aside from gene therapeutic efforts to enhance the transduction efficiency and the membrane targeting of the ChR, discovery and engineering of ChRs with larger single channel current will be valuable activities to further lower the oCI energy budget.

Current preclinical work often involves early postnatal intracochlear injection of adeno-associated virus (AAV) which routinely achieves transduction of ≥70% of the SGNs,^46^^,^^47^^,^^48^ outperforming transduction upon direct intramodiolar pressure injections of AAV in the adult animal (10–⁠40%).^39^^,^^93^ AAV are good candidate vectors, not compromising hearing^96^^,^^97^^,^^98^^,^^99^ and providing long-term transgene expression in postmitotic target cells^47^^,^^100^^,^^101^ despite them not integrating into the host cell genome.^32^ For example, stable expression of ChR over ∼2 years upon administration of a single AAV dose was demonstrated for the mouse SGNs^43^ and stable expression in retinal ganglion cells was shown in for non-human primates.^102^ In general, AAV-based gene delivery is already in use in numerous clinical trials on the eye.^32^^,^^103^ First results of a clinical study on AAV-mediated optogenetic manipulation of retinal ganglion cells show favorable safety data.^104^ Preclinical efforts are being undertaken to develop efficient AAV-based genetic SGN manipulation of the mature cochlea that include work on non-human primates.^105^ Optimized ChRs and their expression in SGNs for energy-efficient optogenetic stimulation is an important requirement for optogenetic hearing restoration.

## Predictions for optical sound coding

Optical sound coding strategies need to consider several constraints such as limited temporal fidelity of coding reflecting ChR kinetics, increased number of stimulation channels relative to electrical CI system, requirements for the power-efficient operation of optical emitters, maximal current consumption, and battery lifetime. The minimal duration and intensity of a light pulse sufficient for optogenetic SGN activation depends on the level of ChR expression (see above), the single channel conductance of the ChR, as well as on the ChR’s closing kinetics. Taking advantage of the expected greater frequency selectivity of the oCI, parallel stimulation by a number of emitters selected from 50 to ≥100 emitters, could be considered, which seems feasible given the current technology. Recruitment of neighboring emitters for increasing the population of activated SGNs at a given tonotopic position will likely help mimic physiological loudness coding that relies on increasing the number of activated SGNs and their firing rate.

### Parallel stimulation

**Figure 5:**
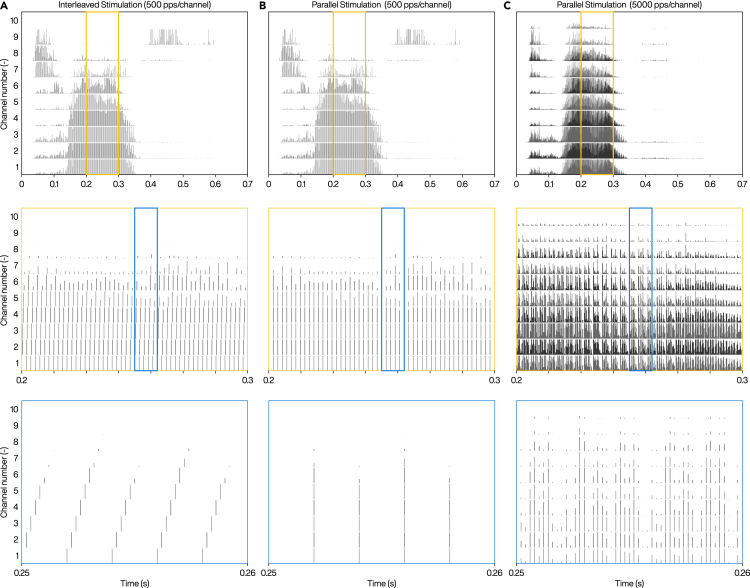
Emittograms representing the output signals of sound coding strategies with different stimulation modes for eCI or oCI (A) Interleaved stimulation at 500 pps/channel. (B) Parallel stimulation at 500 pps/channel. (C) Parallel stimulation at 5000 pps/channel. The top panels show the activation patterns for an audio sample of the word “choice”, and the middle and bottom panels are the zoomed-in plots. The sound processing was based on CIS strategy for ten channels. The vertical lines within the plots represent the onset of the eCI or oCI pulses and not the actual pulses. Parallel stimulation offers longer pulse duration, or higher pulse rate, or a combination of both.

To evaluate advantages of parallel stimulation over interleaved stimulation, we employed custom scripts in MATLAB R2016a (The MathWorks Inc.) to generate electrodograms or “emittograms” for a ten-channel implant, state-of-the-art eCI or e.g., LED-based active oCI used in proof-of-concept studies of oCI system^52^^,^^55^ (Figure 5). Sound processing stages were implemented similar to that of the CIS strategy,^56^ except that there was no interleaving in the parallel stimulation (for details see STAR Methods in supplemental information). For a fixed pulse rate, parallel stimulation offers a longer pulse duration (Figure 5B) in comparison to interleaved stimulation Figure 5A), which is advantageous for oCI systems that accommodate the limited temporal fidelity of optogenetic stimulation with state-of-the-art ChRs (see above). Employing ChRs with faster kinetics (hence increasing temporal fidelity of coding) and larger conductance could enable stimulation at higher rates while keeping the power budget in check (Figure 5C).

### Increased number of emitters

Since the oCI development is still at the preclinical stage, it is not yet possible to test sound coding strategies in studies with human patients. However, *in silico* evaluation approaches will be beneficial for the future development of the entire system and smooth translation into clinical trials. They ideally complement animal experiments and help to focus these studies, thereby reducing the number of animals required for preclinical evidence. Existing speech intelligibility metrics could be used for analysis of *in silico* results (for extensive review and comparison see ref.^106^). In general, most of them are based on the articulation index (AI) framework developed already in 1947 at the Bell Telephone Laboratories for solving problems not related to CIs.^107^ AI allowed for quantitative analysis of the sound recognition capability of human hearing regarding fundamental characteristics of the input sound. The fractional articulation index (fAI)^108^ based on AI, and short-time objective intelligibility measure (STOI)^109^ are the metrics most commonly used to study intelligibility of eCI coding strategies as they are free from prediction bias^106^ and their scores show high correlation with speech intelligibility.^108^^,^^109^ Although both of them could be good candidates for evaluation of oCI coding strategies, for the following analysis we selected fAI which tends to underestimate intelligibility as compared to STOI and, hence, represents a lower boundary of what can be achieved.^106^

**Figure 6:**
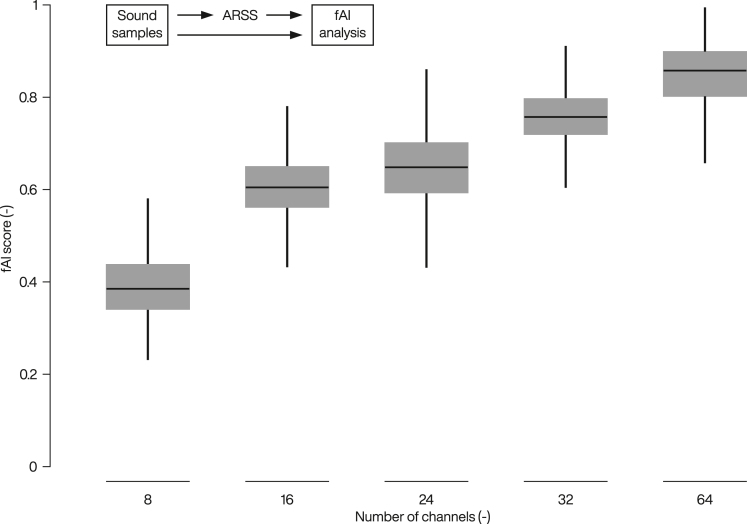
Objective intelligibility measure for different number of spectral channels The fractional articulation index (fAI) score was calculated from comparison of audio resynthesized using Analysis & Resynthesis Sound Spectrograph (ARSS) to the original audio from 10 speakers with 3842 utterances in total. White noise at a signal-to-noise ratio (SNR) of +5 dB was added to each file. Stimulation rate was set to 500 pps/channel. Each boxplot displays the median, the lower and upper quartiles, and the minimum and maximum values that are not outliers. Outliers (not shown) were computed using the interquartile range (points above the upper quartile +1.5 times the distance between upper and lower quartile or below the lower quartile −1.5 times the distance between the upper and lower quartile).

While several studies support that the hearing performance of eCI users improves with increasing number of spectral channels,^8^^,^^110^^,^^111^ a number of studies questioned improvement beyond 10 channels.^7^^,^^8^^,^^9^^,^^10^^,^^11^ The reason for this limitation has been attributed to electrode interactions^10^ as well as CI technology and implantation techniques at the time of these studies.^112^ A prospective oCI with high spectro-temporal resolution is expected to overcome these limitations of CI hearing. In this context, we performed an *in silico* evaluation of speech intelligibility with different number of channels (up to 64 spectral bands). The evaluation was implemented in MATLAB R2016a (The MathWorks Inc.) and used publicly available code of Analysis & Resynthesis Sound Spectrograph (ARSS)^113^ and fAI.^108^ An open speech corpus,^114^ comprising of 10 speakers with 3842 utterances in total, was used for the study (for details see STAR Methods in supplemental information). As the real-world scenarios are rarely in completely quiet environments, we added white noise to each file at a signal-to-noise ratio (SNR) of +5 dB. The ARSS algorithm is based on filter-bank analysis and quite similar to the CIS coding strategy.^56^ The resynthesized audio from ARSS was compared to the original audio by calculating the objective intelligibility measure, fAI. The fAI scores corroborates the hypothesis that higher number of spectral channels is beneficial for speech understanding of CI users, provided there is no channel interaction (Figure 6, where 0 represents poor and 1 high intelligibility). This result is intuitive and supported by the fact that the speech recognition of normal-hearing listeners continues to improve with increasing number of spectral bands, even when the eCI listeners’ performance does not show a significant improvement.^10^ Provided that scaling the number of non-overlapping stimulation channels is feasible for oCI it is expected to offer better speech intelligibility.

Recently, the combined use of electrical and optogenetic stimulation was studied.^41^^,^^42^^,^^43^ Combined sub-threshold optical followed by electrical stimuli resulted in reduction of thresholds beyond those needed for each modality alone. It could be also worth investigating if optogenetic stimulation would benefit from preceding sub-threshold electric stimuli as reported for the INS hybrid approach.^115^ In any case, however, it would require more advanced and elaborate hardware, resulting in technologically more complex CIs with larger form factor of the implant and yet more new implementations of the sound coding strategies that could possibly take advantage of e.g., lowering thresholds for stimulation and reducing overall power budget of the system.

## Toward a clinical system

Pioneering work of Djourno and Eyriès in France inspired House and Doyle in Los Angeles, and just 4 years later, in 1961, two human patients were implanted with the first prototype of a “real” eCI.^116^^,^^117^^,^^118^ Soon, teams in Melbourne (Graham Clark), San Francisco (Robin Michelson and Michael Merzenich), Vienna (Erwin Hochmair, Ingeborg Desoyer, and Kurt Burian), and Paris (Claude Henry Chouard and Patrick MacLeod) joined the race to deliver a commercial eCI system. By the end of the 1980s, the eCI became a standard hearing rehabilitation device.^119^ Now, around 60 years after the first CI implantation, the oCI concept started a similar path and it might be another uphill battle as the oCI must compete with a well-established eCI in terms of hearing quality, stability, risks, and costs to make it attractive to patients and health systems. In this case, not only the device needs to be tested in clinical trials on human and approved but also optogenetic modification of the SGNs via AAV-mediated gene therapy.

However, as the number of physical channels in eCI, as well as speech recognition in quiet reached steady state for over a decade,^6^^,^^120^ it might be a good time to shift focus of engineers toward oCIs. In a German survey study hearing impaired patients (26% bilateral) revealed a demand for improved CI hearing beyond their current experience with eCI, mainly in terms of speech recognition in background noise, greater music appreciation, and more natural sound impression.^15^ Overcoming these shortcomings by increasing the number of channels using oCI and implementation of new sound coding strategies seem worth the effort. As mentioned, substantial parts of the current technology can likely be adapted to work in oCIs. Hence, that focus of oCI development needs to be mostly put on the optical stimulation module and the implant driver (hardware development) and a new sound coding strategy (software development). Given the combination of gene therapy and new medical device the costs of the future oCI system will likely exceed nowadays eCI system (average lifetime cost of €53,000 in case of unilateral implantation^121^). Nevertheless, if achieved, better quality of life by improved speech and music perception could justify the cost difference. Aside from possible benefits for hearing restoration, other factors such as awareness and apprehension of gene therapy, aesthetics, or convenience in terms of battery lifetime will play a role for the patient’s choice, too. Building trust based on good gene therapy education combined with the use of minimal invasive methods should clearly highlight risk-to-benefit ratio of oCI for the future patients.^122^ As the oCI would follow the form of the current eCI, aesthetics important for many patients would not be compromised over eCI systems.^123^ Depending on the final decision on emitter type (active or passive) to be used in the oCI, battery lifetime may vary. Nevertheless, careful design of sound coding strategy in terms of stimulation patterns will help to extend this time. High demand beyond hearing aids and CIs will speed up the development of high-capacity rechargeable batteries.

## Risks and issues of optical cochlear implant technology

The main objective of oCI development is an increased number of non-overlapping stimulation channels inside the cochlea to enable higher spectral resolution than in eCI. Technological feasibility is suggested for active oCIs that could integrate 144 μLEDs on 12 mm (a third of the length of human scala tympani). The insertion of active oCIs into explanted mouse cochlea—5-fold smaller than human cochlea—with 93 μLEDs inside Scala tympani covering a tonotopic frequency range of 72.2 kHz (base) to 2.5 kHz (apex) was reported.^124^ Moreover, functional SGN stimulation by oCI versions with fewer μLEDs was demonstrated in Mongolian gerbils.^28^^,^^50^ Given the roughly 3 times larger human cochlea, it would seem amenable to implant oCIs of similar design.^124^ Yet, the challenge for clinical translation of such active oCIs is the stable encapsulation that provides hermetic sealing of the optoelectronics and yet maintains optical transparency and mechanical flexibility. Also, due to the limited optoelectronic conversion efficiency of LEDs, active oCIs were shown to heat up in their core in the worst-case scenario when LEDs were operated at much higher currents then necessary to elicit behavioral response in rats.^75^ Although, the temperature around these 10-channel oCIs never exceed 1 K at a distance of ∼100 μm (less than the distance of the oCI to the SGNs) which is below 2 K limit of the ISO 14708-1 standard for implantable medical devices, heat dissipation is an important concern. These problems do not plague the waveguide-based passive oCI implementation and this advantage could make them a favorable choice for the first generation of clinical oCIs.

Due to the trade-off between ChR open time and energy requirements, sound coding strategies of the first generation oCIs will likely operate at lower stimulation rates than eCIs. This could compromise the temporal fidelity of sound encoding such as for sound localization, which however, is limited also for eCI (mostly due to the lack of synchronization of stimulation in bilateral eCI). With a vector strength of 0.5 for stimulation rate of ≥200 Hz we would expect the temporal precision of oCI coding to enable temporal fine structure coding, which has been implemented for low frequency eCI channels with subtle improvements in speech understanding.^125^ Encoding the sound envelope has been shown to operate well also with lower eCI rates^126^^,^^127^^,^^128^ that would seem amenable to oCI coding.

In order to meet current standards, oCIs will need to function for many years without failure ideally covering lifespan of patient. In reality, the survival rate of eCIs strongly depends on device and patient age and reaches up to 30 years in a 30-year analysis window with MED-EL devices reaching 10-year cumulative survival rate of 99% in adults and 97% in children.^129^ Due to possible similarities in design between contemporary eCI and future oCI (Figure 3A) similar protection of internal components should be achievable. In such case, lower longevity of the device could be expected mostly due to light-emitting elements failure. In case of LEDs expected lifetime can reach up to 100,000 (depending on construction^130^^,^^131^) while for laser diodes up to 70,000 h (at around 40°C in continuous-wave operation^132^). Assuming continuous stimulation at rate of 300 pps with 1-ms-long pulses the oCI could reach decades-long operation: 40 years for LED or 25 years for laser diodes. Nevertheless, failures of individual emitters are to be expected in decade-long operation. In our unpublished preclinical work, we use oCIs with various extents of μLEDs failures which in most cases where scattered randomly along the length of the oCI. Extended dropouts potentially leading to “dead zones” of stimulation were not observed, but their occurrence during decade-long operation cannot be excluded. This will reduce the oCIs capacity of gapless stimulation of the tonotopic array of SGNs. However, dropouts of electrodes are quite common in clinical eCI and, if individual, can typically be coped with by the patients. Nonetheless, reimplantation is undertaken if electrode array failure is more substantial and this also presents an option with future oCIs.

Longevity of the entire concept of optogenetic hearing restoration also depends on stable expression of ChRs. Unleashing the full potential of optogenetic hearing restoration will be best served with a rich complement of light-sensitive auditory nerve fibers.^43^ Ideally, most if not all of still available SGNs should be transduced. From the vision restoration efforts it seems that 30% of ChR-expressing retinal ganglion cells are realistic and sufficient.^133^ Nevertheless, efforts should be taken to increase the transduction rates by more efficient means of virus administrating. Ideally, a standardized non-invasive method will help assess the functional ChR expression in the SGN e.g., by optical ABR measurements as is heavily used in preclinical studies. In case of insufficient transduction, redosing of AAV could be considered and a procedure has been suggested based on a temporal bone study.^134^ This might also be considered for patients that experience reduced efficiency of oCI function that cannot be fixed by refitting of the oCI.

Another critical point concerning oCI function is the orientation of emitters for optimal optical stimulation. Irradiance at the level of the SGN somata in Rosenthal’s canal was recently investigated as a function of emitter distance and orientation in an *in silico* study of intracochlear light propagation.^44^ Results show that the irradiance follows the inverse-square law of optics when shifting emitter toward or away from the Rosenthal’s canal and emitter orientation has greater impact on irradiance for waveguide-than for μLED-based implants. If waveguide outcoupling with low numerical aperture is chosen, the irradiance in the ganglion decreases with the change of the emitter orientation by ±30° relative to the direct vector from emitter surface to the Rosenthal’s canal. Here, a good balance of efficiency of stimulation and robustness toward axial CI rotation should be considered. Although, most of solutions for preclinical oCI are based on biocompatible materials or at least encapsulated in such, insertion of foreign body into the cochlea will cause grow of a scar tissue. Computational modeling shows an attenuation of irradiance at the SGN somata, but due to forward scattering by scar tissue the spread of excitation was not significantly greater.^44^ Efforts toward hearing preservation surgery and mitigation of the foreign body reaction will help maintaining high efficiency of optical stimulation. Indeed, studies of corticosteroids release in the cochlea have shown potential in decreasing the scar tissue formation.^135^^,^^136^^,^^137^ In terms of design, reliable stimulation with less optical spread might be achieved using perimodiolar implants designed to stay in close proximity to the SGNs.^138^^,^^139^ Preoperative imaging to plan a safe trajectory and robotic insertion are among future techniques that could help oCI to achieve better performance.^140^^,^^141^

Considering energy consumption, a custom-built preclinical CI system managed to achieve similar time of operation of single-channel stimulation with eCI and oCI based on μLEDs (8 vs. 7 h, respectively) using general purpose commercial out of shelf components.^55^ Assuming twice as many channels for oCI then eCI, it would result in half of the battery life for oCI comparing to eCI. By using dedicated components and optimization of the system for clinical use this should still stay comparable. Nevertheless, a waveguide-based oCI system and f-Chrimson^47^ as the ChR considering coding strategies compared to current eCIs would require a 2-fold greater energy consumption. This could be improved by development of less power-hungry laser diodes and/or ChRs with 5–10-times larger conductance that have been recently described.^142^^,^^143^

## Supplemental information

Document S1. STAR Methods
