# Supplementary material for: En route to sound coding strategies for optical cochlear implants
Source: iScience. 2023 Aug 25;26(10):107725. doi: 10.1016/j.isci.2023.107725 (PMC10502376; doi:10.1016/j.isci.2023.107725)
Supplement: Document S1. STAR Methods [file mmc1.pdf]

iScience, Volume *26*

Supplemental information

En route to sound coding strategies for optical cochlear implants

Lakshay Khurana, Tamas Harczos, Tobias Moser, and Lukasz Jablonski

STAR Methods

RESOURCE AVAILABILITY

Lead contact

Further information and requests for resources should be directed to and will be fulfilled by the lead contact, Lukasz Jablonski (lukasz.jablonski@wp.eu).

Materials availability

This study did not generate new unique materials.

Data and code availability

- Data used as the input for frameworks of this study (Figure 5 and Figure 6) is publicly available. Identifiers are listed in the key resources table. Data reported in this study (Figure 5 and Figure 6) is available from lead contact upon request.
- Code and/or algorithms used in frameworks of this study (Figure 5 and Figure 6) are publicly available. DOIs and/or other identifiers are listed in the key resources table. All original code is available from lead contact upon request.
- Any additional information required to reanalyse the data reported in this paper is available from the lead contact upon request.

METHOD DETAILS

For evaluation of parallel stimulation (simultaneous presentation of stimuli) that could be enabled by oCI in comparison to interleaved stimulation (non-simultaneous presentation of stimuli) typical for eCI, electrodograms or "emittograms" for a ten-channel eCI or oCI were generated using a framework established in MATLAB R2016a (The MathWorks Inc.; **Figure 5**). As the activation pattern an audio sample of the single word "choice" spoken by a female speaker was used. The sound processing in the input of the framework was based on CIS strategy[56] implemented for ten channels that could represent state-of-the-art eCI or e.g. LED-based active oCI used in proof-of-concept studies of oCI system[52,55]. Stimulation pattern in the output stage of the framework was either interleaved at 500 pps/channel (**Figure 5 A**), parallel at 500 pps/channel (**Figure 5 B**), or parallel at 5000 pps/channel (**Figure 5 C**). Emittograms show vertical lines representing the onset of the eCI or oCI pulses and not the actual pulses so that visualisation is independent of stimulation driver.

For evaluation of increased number of independent channels another framework established in MATLAB R2016a (The MathWorks Inc.) was used (**Figure 6**). As an input to the framework open speech corpus[114], comprising of 10 speakers with 3842 utterances in total, was used with addition of a white noise to each file at a signal-to-noise ratio (SNR) of +5 dB to get closer to the real-world scenarios where completely quiet environments are rare. The first stage of the framework used a code of Analysis & Resynthesis Sound Spectrograph (ARSS)[113] The filter-bank analysis of the ARSS algorithm[113] is based on the CIS coding strategy[56] with a stimulation rate set to 500 pps/channel. Here, the original audio is first filtered using a filter bank consisting of overlapping logarithmic-scale frequency-domain Hann window functions. Next, envelope detection is performed on filtered signals to obtain magnitude of the analytic signal. Resulting envelope for each frequency band is

represented by a horizontal line at different frequencies of a spectrogram image and each envelope amplitude by the line intensity. For the resynthesis of a sound from spectrogram each horizontal line representing central frequency is upsampled to the final signal sampling rate and is then modulated with sine wave matching its central frequency. The last stage of the framework, in which audio resynthesised by ARSS was compared to the original input audio, was based on fractional articulation index fAI[108] providing objective intelligibility measure where 0 represents poor and 1 high intelligibility.

KEY RESOURCES TABLE

REAGENT or RESOURCE	SOURCE	IDENTIFIER
Deposited data		
Raw data; Free ST American English Corpus (sound samples)	OpenSLR (SLR45); ST-AEDS-20180100_1, Free ST American English Corpus[114]	https://www.openslr.org/45/
Software and algorithms		
Analysis & Resynthesis Sound Spectrograph (ARSS)	Rouzic[113]	https://arss.sourceforge.net/
Fractional articulation index (fAI)	Loizou and Ma[108]	https://doi.org/10.1121/1.3605668
Continuous interleaved sampling (CIS)	Wilson et al.[56]	https://doi.org/10.1038/352236a0
